# Phosphorylation of estrogen receptor α serine 167 is predictive of response to endocrine therapy and increases postrelapse survival in metastatic breast cancer

**DOI:** 10.1186/bcr1285

**Published:** 2005-07-27

**Authors:** Hiroko Yamashita, Mariko Nishio, Shunzo Kobayashi, Yoshiaki Ando, Hiroshi Sugiura, Zhenhuan Zhang, Maho Hamaguchi, Keiko Mita, Yoshitaka Fujii, Hirotaka Iwase

**Affiliations:** 1 Oncology and Immunology, Nagoya City University Graduate School of Medical Sciences, Nagoya, Japan; 2 Oncology and Endocrinology, Nagoya City University Graduate School of Medical Sciences, Nagoya, Japan; 3 Josai Municipal Hospital of Nagoya, Nagoya, Japan

## Abstract

**Introduction:**

Endocrine therapy is the most important treatment option for women with hormone-receptor-positive breast cancer. The potential mechanisms for endocrine resistance involve estrogen receptor (ER)-coregulatory proteins and crosstalk between ER and other growth factor signaling networks. However, the factors and pathways responsible for endocrine resistance are still poorly identified.

**Methods:**

Using immunohistochemical techniques, we focused on the expression and phosphorylation of hormone receptors themselves and examined the phosphorylation of ER-α Ser118 and ER-α Ser167 and the expression of ER-α, ER-β1, ER-βcx/β2, progesterone receptor (PR), PRA, and PRB in the primary breast carcinomas of 75 patients with metastatic breast cancer who received first-line treatment with endocrine therapy after relapse.

**Results:**

Phosphorylation of ER-α Ser118, but not Ser167, was positively associated with overexpression of HER2, and HER2-positive tumors showed resistance to endocrine therapy. The present study has shown for the first time that phosphorylation of ER-α Ser167, but not Ser118, and expression of PRA and PRB, as well as ER-α and PR in primary breast tumors are predictive of response to endocrine therapy, whereas expression of ER-β1 and ER-βcx/β2 did not affect response to the therapy. In addition, patients with either high phosphorylation of ER-α Ser167, or high expression of ER-α, PR, PRA, or PRB had a significantly longer survival after relapse.

**Conclusion:**

These data suggest that phosphorylation of ER-α Ser167 is helpful in selecting patients who may benefit from endocrine therapy and is a prognostic marker in metastatic breast cancer.

## Introduction

The development and progression of breast cancer are influenced by steroid hormones, particularly estrogen, via their interaction with specific target receptors. Endocrine therapy has become the most important treatment option for women with estrogen receptor (ER)-positive breast cancer. Nevertheless, many breast cancer patients with tumors expressing high levels of ER are unresponsive to endocrine therapy, and all patients with advanced disease eventually develop resistance to the therapy. The potential mechanisms behind either this intrinsic or acquired endocrine resistance involve ER-coregulatory proteins and crosstalk between the ER pathway and other growth factor signaling networks [1,2]. An understanding of the molecular mechanisms that modulate the activity of the estrogen signaling network has enabled new ways of overcoming endocrine resistance to be developed.

ER-α is phosphorylated on multiple amino acid residues [3]. Serines 104, 106, 118, and 167 are all located within the activation function (AF)1 region of ER-α, and their phosphorylation provides the important mechanism that regulates AF1 activity [4,5]. In response to estradiol binding, human ER-α is phosphorylated mainly on Ser118 and to a lesser extent on Ser104 and Ser106 [4]. Although some authors have also reported that Ser167 is a major estradiol-induced phosphorylation site [5,6], this response to estradiol has not been universally observed [4,7]. Interestingly, in response to the activation of the mitogen-activated protein kinase (MAPK) pathway, phosphorylation occurs on Ser118 and Ser167 [8,9]. However, the role of phosphorylation of Ser118 and Ser167 of ER-α in human breast cancer has not been investigated.

ER-β and its splicing isoforms are widely expressed in both normal and malignant breast tissue [10]. Although several groups have reported results regarding the possible function of ER-β, and its potential as a prognostic or predictive factor in breast cancer, the data remain inconclusive and are often contradictory [11,12]. ER-βcx (also called ER-β2), a splice variant of ER-β, is considered to be a dominant repressor of ER-α; it is identical to ER-β1 (wild-type ER-β) except that the last exon, 8, is replaced by 26 amino acid residues [13]. The role of ER-β and its isoforms, especially with respect to the response of breast cancer to endocrine therapy, has also not been elucidated.

Progesterone receptors (PRs) occur as two isoforms, PRA and PRB, transcribed from two distinct promoters on a single gene. PRA, but not PRB, lacks the 164 amino acid N-terminal residues that contain AF3, and this is the cause of their functional differences [14]. In the mammary gland, the overexpression of PRA relative to PRB results in extensive epithelial cell hyperplasia, excessive ductal branching, and a disorganized basement membrane, all features associated with neoplasia [15]. In contrast, the overexpression of PRB leads to premature arrest of ductal growth and inadequate lobuloalveolar differentiation [16]. However, little is known about the unique roles of the two PR isoforms in breast cancer.

In this study, we focused on the expression and phosphorylation of the hormone receptors themselves and, using immunohistochemistry (IHC), examined the phosphorylation of ER-α Ser118 and Ser167 and the expression of ER-α, ER-β1, ER-βcx/β2, PR, PRA, and PRB in primary breast tumor specimens from 75 patients with metastatic breast cancer who received first-line treatment with endocrine therapy on relapse. Our results show that patients with primary breast tumors in which there is either high phosphorylation of ER-α Ser167 or high expression of ER-α, PR, PRA, or PRB significantly responded to endocrine therapy and had a better survival after relapse.

## Materials and methods

### Cell culture and transfections

COS-7 cells (ATCC American Type Culture Collection, Manassas, VA, USA) were grown in DMEM containing 10% fetal calf serum, 2 mM L-glutamine, and penicillin–streptomycin (50 IU/ml and 50 mg/ml, respectively) at 37°C with 5% CO_2 _as described previously [17]. T47D cells (ATCC) were grown in RPMI-1640 supplemented with 10% fetal calf serum, 2 mM L-glutamine, and penicillin–streptomycin (50 IU/ml and 50 mg/ml, respectively), at 37°C with 5% CO_2_. Six microliters of FuGENE6 transfection reagent (Roche Molecular Biochemicals, Indianapolis, IN, USA), 3 μg of an expression vector for human ER-α cDNA (pSG5/puromycine hERα, full length, kindly provided by Pierre Chambon, Strasbourg, France) were used for transfection into COS-7 cells as described previously [18]. After transfection, cells were starved in serum-free DMEM without phenol red for 20 hours.

### Immunoblotting

Cells were treated in the absence or presence of 17β-estradiol (E_2_) (10 nM, Sigma-Aldrich Co, St Louis, MO, USA) and/or epidermal growth factor (EGF) (100 ng/ml, human recombinant EGF, Sigma-Aldrich) for 30 min, pelleted by centrifugation and solubilized in lysis buffer containing 10 mM Tris-HCl, pH 7.6, 5 mM EDTA, 50 mM NaCl, 30 mM sodium pyrophosphate, 50 mM sodium fluoride, 1 mM sodium orthovanadate, 1% Triton X-100, 1 mM phenylmethylsulfonylfluoride (PMSF), 5 μg/ml aprotinin, 1 μg/ml pepstatin A, and 2 μg/ml leupeptin, as described previously [19]. Equal amounts of total protein from whole-cell lysates were prepared and used for SDS–PAGE. Immunoblotting was performed as described previously [18] using polyvinylidene difluoride (PVDF) membranes (Invitrogen, Carlsbad, CA, USA; Catalogue no. LC2002), and polyclonal antibody against ER-α (H-184, Santa Cruz Biotechnology, Santa Cruz, CA, USA) (1:200 dilution), polyclonal rabbit antiphospho-ER-α (Ser118) antibody (Cell Signaling Technology, Beverly, MA, USA) (1:500 dilution), polyclonal rabbit antiphospho-ER-α (Ser167) antibody (Cell Signaling) (1:500 dilution) as primary antibodies and horseradish-peroxidase-conjugated goat antibodies to rabbit IgG as secondary antibodies in conjunction with enhanced chemiluminescence substrate mixture (SuperSignal WestPico Chemiluminescent Substrate, Pierce, Rockford, IL, USA) in accordance with the manufacturer's instructions. Phospho-ER-α Ser118 antibody detects ER-α only when the receptor is phosphorylated at Ser118, and not at Ser106 or Ser167; and phospho-ER-α Ser167 antibody detects ER-α only when the receptor is phosphorylated at Ser167, and not at Ser106 or Ser118, by immunoblotting, as described by Chen and colleagues [20].

### Generation of specific antibodies for ER-β proteins

To detect specific ER-β1 and ER-βcx/β2 proteins, rabbit polyclonal antibodies were generated against synthesized peptides of the C-terminal region of ER-β1 (CSPAEDSKSKEGSQNPQSQ) and ER-βcx/β2 (MKMETLLPEATMEQ), in accordance with the method of Ogawa and colleagues [13] and purified on affinity columns bound with each synthetic peptide as described previously [17]. To confirm the specificity of these polyclonal antibodies, immunoblot analysis was performed using COS-7 cells transfected with either expression plasmid encoding ER-β1 or ER-βcx/β2 (kindly donated by Masami Muramatsu, Saitama, Japan) as previously described [18]. Immunoblotting with specific anti-ER-β antibodies showed that the polyclonal antibody for ER-β1 detected a specific band at 60 kDa only in the lysates of COS-7 cells transfected with an ER-β1 expression plasmid, and not in those transfected with an ER-βcx/β2 expression plasmid, as described previously by Ogawa and colleagues [13]. Conversely, the polyclonal antibody for ER-βcx/β2 detected a specific band at 55 and 51 kDa only in the lysates of COS-7 cells transfected with an ER-βcx/β2 expression plasmid, and not in those transfected with an ER-β1 expression plasmid, as described previously by Ogawa and colleagues [13].

### Patients and breast cancer tissues

Breast tumor specimens from 75 women with metastatic breast cancer who were treated at Nagoya City University Hospital between 1982 and 2002 were included in this study (Table 1). The study protocol was approved by the institutional review board and conformed with the guidelines of the 1975 Declaration of Helsinki. All patients had undergone surgical treatment for primary breast cancer (either mastectomy or lumpectomy) and all primary tumors were ER^+ ^or PR^+^. After surgery, five patients (6.7%) received no additional therapy. Of the remaining 71 patients, 32 (42.7%) received systemic adjuvant therapy consisting of endocrine therapy (tamoxifen) alone, two (2.7%) received chemotherapy alone, and 36 (48%) received combined endocrine therapy and chemotherapy. Patients who were positive for axillary lymph nodes received either oral administration of 5-fluorouracil derivatives for 2 years or a combination of cyclophosphamide, methotrexate, and fluorouracil (CMF). Patients were observed for disease recurrence at least once every six months for the first 5 years after the surgery and once every year thereafter.

### First-line endocrine therapy for metastatic breast cancer and response criteria

When the patients relapsed and were diagnosed with metastatic breast cancer, they started endocrine therapy (Table 1). Patients were assessed monthly for clinical response, which was defined according to World Health Organization criteria as complete response, partial response, no change, or progressive disease. The presence of progressive disease indicated treatment failure; all other clinical responses were considered to show efficacy of treatment.

### Immunohistochemical analysis

One 4-μm section of each submitted paraffin block was stained first with hematoxylin and eosin to verify that an adequate number of invasive carcinoma cells were present and that the fixation quality was adequate for immunohistochemical (IHC) analysis. Serial sections (4 μm) were prepared from selected blocks and float-mounted on adhesive-coated glass slides, for ER-α, ER-β, and PR staining as described previously [21]. Primary antibodies included monoclonal mouse antihuman ER-α antibody (1D5, DAKO, Glostrup, Denmark) at 1:100 dilution for ER-α; polyclonal rabbit antiphospho-ER-α (Ser118) antibody (Cell Signaling) at 1:25 dilution for phosphorylated ER-α Ser118; polyclonal rabbit antiphospho-ER-α (Ser167) antibody (Cell Signaling) at 1:50 dilution for phosphorylated ER-α Ser167; polyclonal rabbit anti-ER-β1 antibody at 1:10000 dilution for ER-β1; polyclonal rabbit anti-ER-βcx/β2 antibody at 1:2000 dilution for ER-βcx/β2; monoclonal mouse antihuman PR antibody (636, DAKO) at 1:100 dilution for PR; monoclonal mouse antihuman PR antibody (Ab-7, Neo Markers, Fremont, CA) at 1:100 dilution for PRA; and monoclonal mouse antihuman PR antibody (Ab-2, Neo Markers) at 1:100 dilution for PRB. With respect to the PRA and PRB antibodies, it has been reported that whereas AB-7 can recognize high-PRA and low-PRB forms, this antibody recognizes PRA only in 10% formalin-fixed and paraffin-embedded tissue sections, and AB-2 recognizes exclusively PRB in these same media [22]. The DAKO Envision system (DAKO EnVision labelled polymer, peroxidase) was used as the detection system as described previously [21]. HER2 immunostaining was done and evaluated using a method similar to the HercepTest (DAKO) [21].

### Immunohistochemical scoring

Immunostained slides were scored after the entire slide had been evaluated by light microscopy. The expression and phosphorylation of hormone receptors were scored by assigning proportion and intensity scores, in accorance with the procedure of Allred and colleagues [23]. In brief, a proportion score represented the estimated proportion of tumor cells staining positive, as follows: 0 (none); 1 (<1/100); 2 (1/100 to 1/10); 3 (>1/10 to 1/3); 4 (>1/3 to 2/3); and 5 (>2/3). Any brown nuclear staining in invasive breast epithelium counted towards the proportion score. An intensity score represented the average intensity of the positive cells, as follows: 0 (none); 1 (weak); 2 (intermediate); and 3 (strong). The proportion and intensity scores were then added to obtain a total score, which could range from 0 to 8.

### Statistical analysis

The Mann–Whitney *U *test or the Kruskal–Wallis test was used to compare the IHC scores of hormone receptors with clinicopathological characteristics. The Mann–Whitney *U *test and the unpaired *t*-test were used to compare the IHC scores of hormone receptors with response to endocrine therapy. The Spearman rank correlation test was used to study relations between expression and phosphorylation of hormone receptors and disease-free interval. To examine the change of expression and phosphorylation status between the primary and recurrent tumors, the one-sample Wilcoxon signed rank test was used. Estimation of overall survival was performed using the Kaplan–Meier method, and differences between survival curves were assessed with the log-rank test. Cox's proportional hazards model was used for univariate and multivariate analyses of prognostic values.

## Results

### Phosphorylation of ER-α Ser118 and ER-α Ser167 is induced in response to EGF

**Figure 1:**
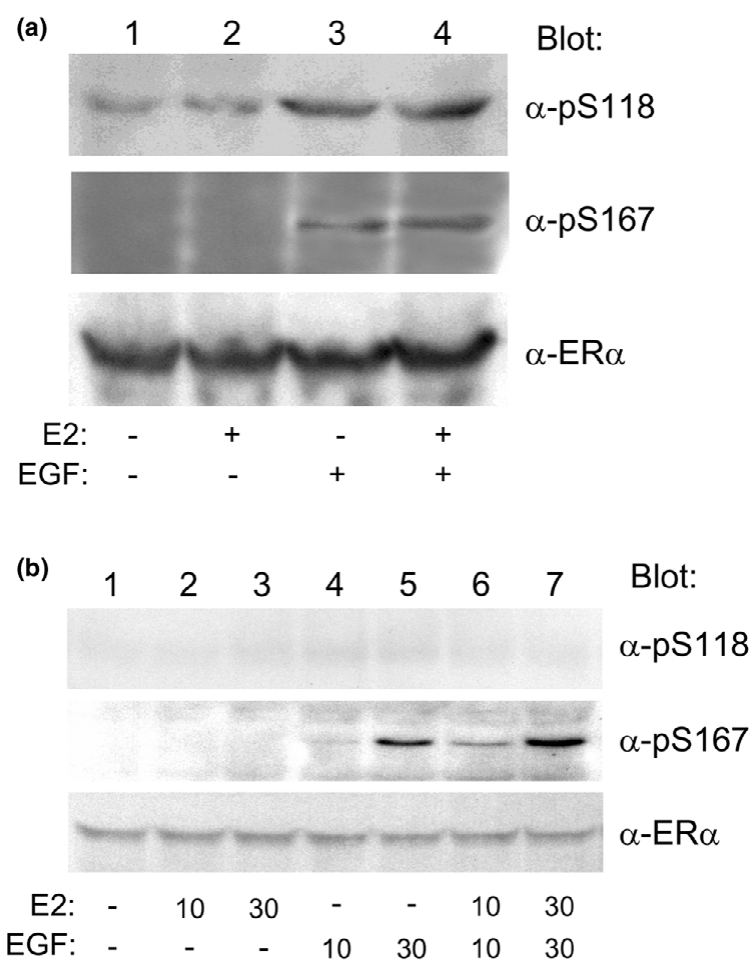
Immunoblot analysis of phosphorylated estrogen receptor (ER)-α Ser118 and ER-α Ser167. **(a) **Transfected COS-7 cells were grown in serum- and estrogen-deprived conditions and treated with vehicle (medium) (lane 1), 17β-estradiol (E_2_) (lane 2), epidermal growth factor (EGF) (lane 3), or E_2 _and EGF (lane 4) for 30 min. Equal amounts of total protein from whole cell lysates were blotted for either anti-ER-α-phosphoserine (α-pS118 and α-pS167) and anti-ER-α (α-ER-α) antibodies. **(b) **T47D cells were grown in serum- and estrogen-deprived conditions and treated with vehicle (medium) (lane 1), 17β-estradiol (E_2_) for 10 min (lane 2) and 30 min (lane 3), EGF for 10 min (lane 4) and 30 min (lane 5), or E_2 _and EGF for 10 min (lane 6) and 30 min (lane 7). Equal amounts of total protein from whole cell lysates were blotted for either anti-ER-α-phosphoserine (α-pS118 and α-pS167) and anti-ER-α (α-ER-α) antibodies.

To test the ability of site-specific antiphosphoserine antibodies for ER-α Ser118 and ER-α Ser167, we examined the phosphorylation status of these two serines in transfected COS-7 cells by immunoblotting. Cells were grown in serum- and estrogen-deprived conditions and treated with vehicle (medium) (Fig. 1a, lane 1), E_2 _(lane 2), EGF (lane 3), or E_2 _and EGF (lane 4). Immunoblotting of replicate samples with antiphohphoserine antibodies showed that ER-α was constitutively phosphorylated on Ser118 (Fig. 1a, lane 1, top panel), but not on Ser167 (Fig. 1a, lane 1, second panel). ER-α became inducibly phosphorylated on both residues Ser118 and Ser167 in response to EGF (Fig. 1a, lanes 3 and 4, top and second panels). On the other hand, phosphorylation of Ser118 and Ser167 was not affected with E_2 _treatment in our analysis (Fig. 1a, lane 1 vs 2, lane 3 vs 4, top and second panels). Expression of ER-α was observed equally in each condition (Fig. 1a, bottom panel).

To further validate the ability of site-specific antibodies for phospho-ER-α Ser118 and Ser167 in ER^+ ^and PR^+ ^breast cancer cells, phosphorylation of ER-α Ser118 and Ser167 was analyzed in T47D cells. Cells were grown in serum- and estrogen-deprived conditions and treated with vehicle (medium) (Fig. 1b, lane 1), E_2 _for 10 min (lane 2) and 30 min (lane 3), EGF for 10 min (lane 4) and 30 min (lane 5), or E_2 _and EGF for 10 min (lane 6) and 30 min (lane 7). ER-α was inducibly phosphorylated on Ser167 in response to EGF (Fig. 1b, lanes 1, 4, 5, 6, and 7, second panel). Phosphorylation of Ser167 was increased with EGF treatment for 30 min compared with that for 10 min (lane 5 vs 4, lane 7 vs 6). On the other hand, ER-α was not phosphorylated on Ser167 in response to E_2 _(Fig. 1b, lanes 2 and 3, second panel). In addition, ER-α was not phosphorylated on Ser118 in response to either E_2 _or EGF (Fig. 1b, lanes 2 to 7, top panel) in T47D cells. Expression of ER-α was observed equally under both conditions (Fig. 1b, bottom panel). We concluded from immunoblotting that, in response to EGF in COS-7 and T47D cells, ER-α Ser167 was inducibly rather than constitutively phosphorylated, and that the phosphorylation of ER-α Ser118 was constitutive and further induced by EGF in COS-7 cells, but that Ser118 phosphorylation was not observed after the stimulation of T47D cells by either E_2 _or EGF.

### Immunohistochemical staining for phosphorylation of ER-α Ser118 and ER-α Ser167, and expression of ER-β 1, ER-βcx/β2, PRA, and PRB in human breast cancer

**Figure 2:**
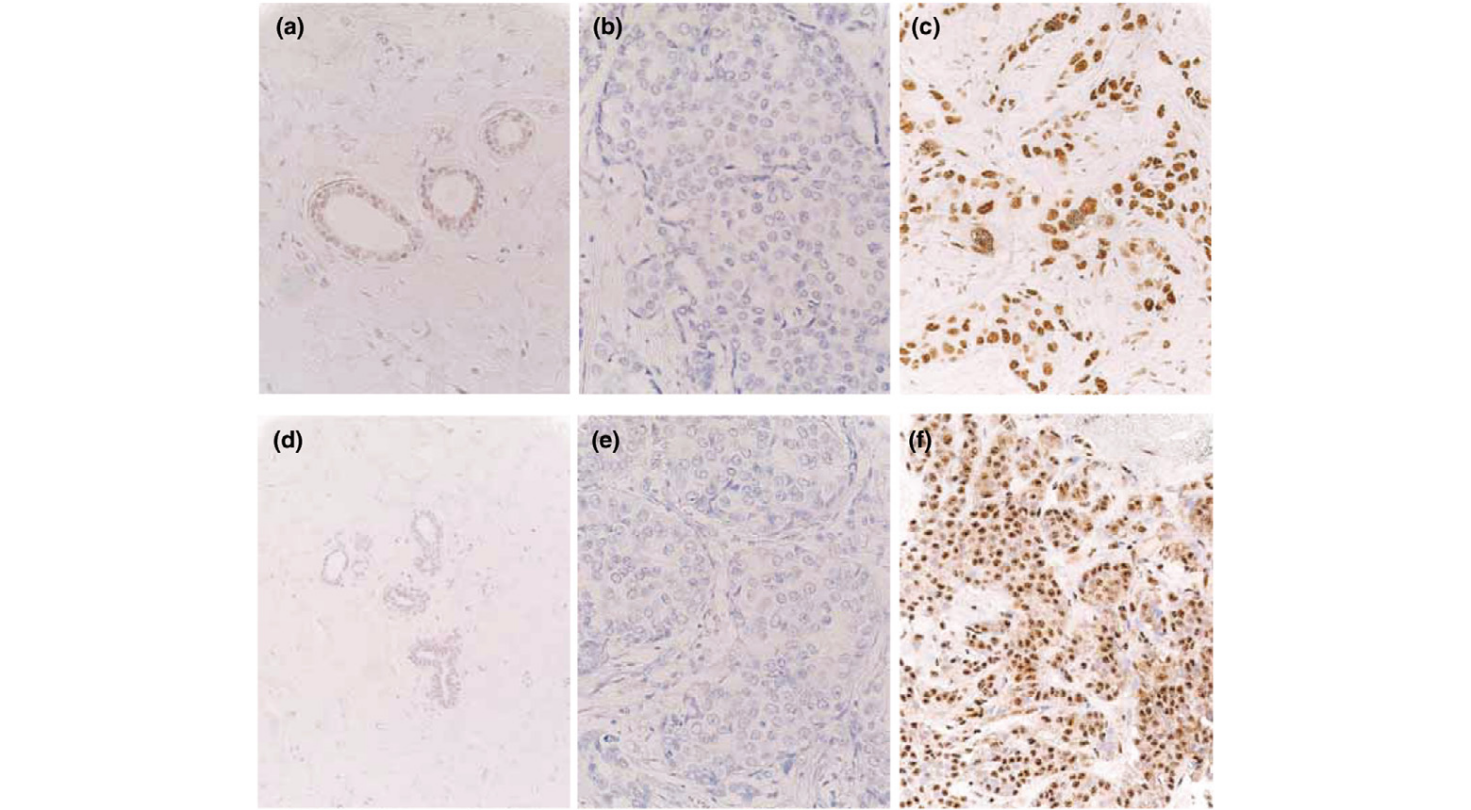
Representative immunohistochemical staining of estrogen receptor (ER)-α Ser118, and ER-α Ser167 in normal breast epithelium and invasive ductal carcinoma. Phosphorylation of ER-α Ser118 in **(a) **normal breast epithelium and **(b,c) **invasive ductal carcinoma: negative (b) and positive (c) nuclear staining was observed in carcinoma cells. Phosphorylation of ER-α Ser167 in **(d) **normal breast epithelium and **(e,f) **invasive ductal carcinoma: negative (e) and positive (f) nuclear staining was observed in carcinoma cells (magnification, 400×).

**Figure 3:**
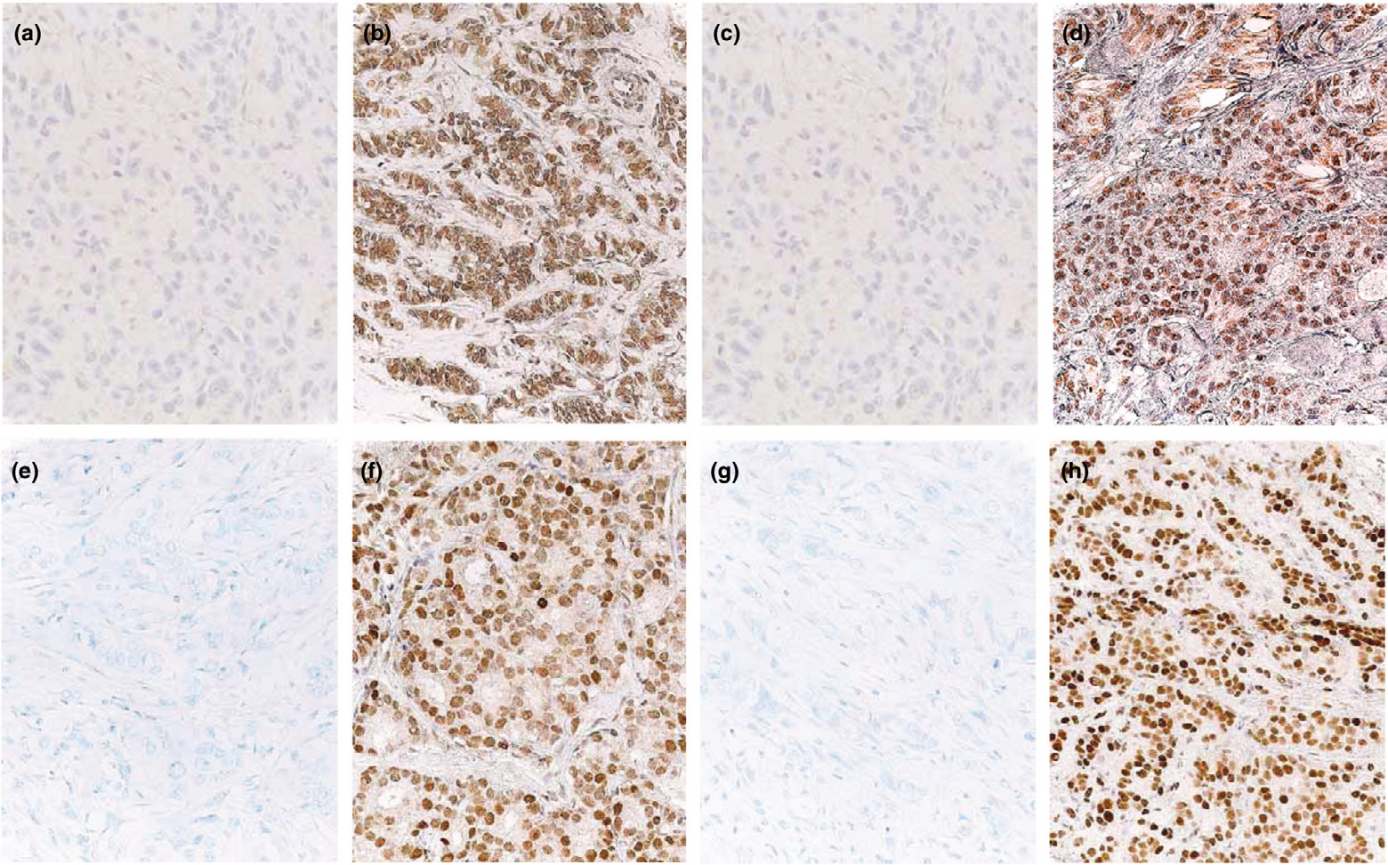
Representative immunohistochemical staining of estrogen receptor (ER)-β1 **(a,b)**, ER-βcx/β2 **(c,d)**, progesterone receptor (PR)A **(e,f)**, and PRB **(g,h) **in invasive ductal carcinoma. Negative (a,c,e,g) and positive (b,d,f,h) nuclear staining was observed in these cells.

To investigate the phosphorylation of ER-α Ser118 and ER-α Ser167 in human breast cancer specimens, IHC analysis was performed using the same site-specific antiphosphoserine antibodies served in the immunoblotting. IHC for phosphorylated ER-α Ser118 (Fig. 2a) and ER-α Ser167 (Fig. 2d) showed presence of nuclear staining in some but not all cells of normal breast epithelium (Fig. 2). Cancer cell nuclei of invasive carcinoma tissues were positively stained with specific antibodies for phosphorylated ER-α Ser118 (Fig. 2c) and ER-α Ser167 (Fig. 2f). Specific detection of expression of ER-β1 (Fig. 3a), ER-βcx/β2 (Fig. 3b), PRA (Fig. 3c), and PRB (Fig. 3d) showed positive nuclear staining in carcinoma cells.

### Correlation between expression and phosphorylation of ER-α, ER-β, and PR and clinicopathological factors in primary breast tumors

We examined the phosphorylation of ER-α Ser118 and Ser167, and expression of ER-α, ER-β1, ER-βcx/β2, PR, PRA, and PRB in 75 primary invasive breast carcinomas by IHC. The IHC scores for ER-α, ER-β, and PR were compared among patient subgroups, according to clinicopathological factors. Phosphorylation of ER-α Ser118, but not of ER-α Ser167, was positively correlated with expression levels of HER2 (*P *= 0.038), whereas expression of ER-α (1D5) tended to be inversely correlated with HER2 overexpression. PR (636) expression was significantly associated with age (*P *= 0.0018). There was no difference between the expression and phosphorylation of hormone receptors and other clinicopathological factors.

### Correlation between expression and phosphorylation of ER-α, ER-β, and PR in primary breast tumors

Links between the IHC scores for the expression and phosphorylation of hormone receptors were analyzed using the Spearman's rank correlation test (Table 2). Phosphorylation of ER-α Ser118 was strongly and positively associated with phosphorylation of ER-α Ser167 (*P *< 0.0001) and with expression of ER-β1 (*P *< 0.0001), ER-βcx/β2 (*P *< 0.0001), and PRA (*P *< 0.0001). Phosphorylation of ER-α Ser167 was also positively correlated with expression of ER-β1 (*P *= 0.0003), ER-βcx/β2 (*P *< 0.0001), and PRA (*P *= 0.0007), whereas no association was found between phosphorylation of ER-α Ser118/Ser167 and expression of ER-α (1D5). A significant correlation was observed between expression levels of ER-β1 and ER-βcx/β2 (*P *< 0.0001). Expression of ER-βcx/β2, but not ER-β1, was positively correlated with expression of PRA (*P *= 0.0011) and PRB (*P *= 0.0052). Strong associations were found between expression of PR (636), PRA, and PRB (*P *< 0.0001, respectively). Expression of ER-α (1D5) was significantly correlated with expression of PR (636) (*P *= 0.0001) and PRA (*P *= 0.028), but not with PRB.

### Phosphorylation of ER-α Ser167, but not Ser118, and expression of PRA and PRB in primary breast tumors are predictive of response to endocrine therapy in metastatic breast cancer

At relapse, all patients received endocrine therapy as first-line treatment for metastatic breast cancer; 35 (46.7%) patients responded. We analyzed whether the expression and phosphorylation levels of hormone receptors in the primary breast tumors affected the response to endocrine therapy when given in this circumstance (Table 3). Patients with primary breast tumors that had high phosphorylation of ER-α Ser167, or high expression of ER-α (1D5), PR (636), PRA, or PRB, significantly responded to endocrine therapy (Mann-Whitney *U *test, *P *= 0.033, *P *= 0.0045, *P *= 0.0008, *P *= 0.0001, and *P *= 0.013, respectively). Interestingly, these patients with primary breast tumors with high phosphorylation or expression of the above factors also had a longer disease-free interval (compare Tables 3 and 4). In contrast, phosphorylation of ER-α Ser118 or expression of ER-β1 or ER-βcx/β2 did not affect response to endocrine therapy. In the subgroup with HER2 overexpression (*n *= 12), phosphorylation of ER-α Ser118 (IHC scores ≥3) was observed in all cases except one, whereas phosphorylation of ER-α Ser167 (IHC scores ≥3) was found in only six. Furthermore, endocrine therapy was effective in only three (25%) patients in the HER2-positive group (data not shown).

### Correlation between expression and phosphorylation of ER-α, ER-β, and PR in primary breast tumors and disease-free interval

We then examined whether the expression and phosphorylation levels of hormone receptors in primary breast tumors affected disease-free interval in relapsing breast cancer patients. Spearman correlation coefficients between the IHC scores of ER-α, ER-β, and PR and disease-free interval are shown in Table 4. The time to relapse after primary surgery was significantly longer in patients with primary breast tumors with high phosphorylation levels of ER-α Ser167 or with high expression levels of ER-α (1D5), PR (636), PRA, or PRB (*P *= 0.0076, *P *= 0.035, *P *= 0.018, *P *= 0.0061, and *P *= 0.023, respectively). On the other hand, no significant relation was observed between either phosphorylation of ER-α Ser118 or expression of ER-β1 or ER-βcx/β2 and disease-free interval.

### Comparison of IHC scores of ER-α, ER-β, and PR in primary and secondary tumors

Biopsy specimens were obtained from the secondary tumors of 10 patients after relapse. Six were local skin or subcutaneous tumors, two were axillar or supraclavicular lymph nodes, and two were distant (lung) tumors. Phosphorylation of ER-α Ser118 was much higher in secondary than in primary tumors (*P *= 0.0098) (Table 5). There was also a tendency for phosphorylation of ER-α Ser167 to increase in secondary tumors, although this did not reach significance. Furthermore, the IHC scores of ER-β1, ER-βcx/β2, PRA, and PRB were all significantly higher in secondary than in primary tumors (*P *= 0.041, *P *= 0.049, *P *= 0.018, and *P *= 0.027, respectively), while expression levels of ER-α (1D5) and PR (636) were lower in secondary than in primary tumors.

### Patients with high phosphorylation of ER-α Ser167 and high expression of PRA and PRB in primary breast tumors had a significantly longer survival after relapse

**Figure 4:**
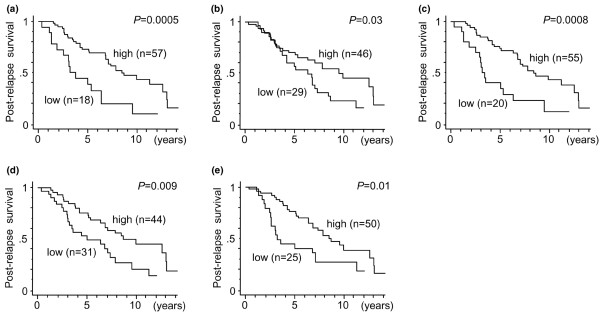
Postrelapse survival of patients analyzed according to expression of **(a) **estrogen receptor (ER)-α, **(b) **phosphorylation of ER-α Ser167, and **(c) **expression of progesterone receptor (PR), **(d) **PRA, and **(e) **PRB. Higher levels of expression or phosphorylation of these factors were associated with a better survival.

Finally, we analyzed whether the expression and phosphorylation levels of hormone receptors in the primary breast tumors affected the survival after relapse. The median follow-up period was 77 months (range, 4 to 234 months). High expression of ER-α (1D5) (IHC score ≥3) significantly increased postrelapse survival (*P *= 0.0005) (Fig. 4a). Patients with high phosphorylation of ER-α Ser167 (IHC score ≥2) had a significant longer postrelapse survival (*P *= 0.03) (Fig. 4b). Similarly, high expression of PR (IHC score ≥3), PRA (IHC score ≥4), and PRB (IHC score ≥3) significantly increased postrelapse survival (*P *= 0.0008, *P *= 0.009, and *P *= 0.01, respectively) (Fig. 4c,d,e). Univariate analysis (Table 6) revealed significant associations between postrelapse survival and phosphorylation of ER-α Ser167 (*P *= 0,035), as well as expression of ER-α (1D5) (*P *= 0,0009), PR (636) (*P *= 0,0012), PRA (*P *= 0,03), and PRB (*P *= 0,013). On the other hand, phosphorylation of ER-α Ser118 and expression of ER-β1 and ER-βcx/β2 did not affect postrelapse survival. The status of phosphorylation of ER-α Ser167, expression of ER-α (1D5), and PRB were selected for the multivariate analysis because these three factors were significant in the univariate analysis and no significant association was found between IHC scores of these factors. Patients with primary tumors with high expression of ER-α (1D5) had significantly increased overall survival (*P *= 0.0076), whereas phosphorylation of ER-α Ser167 or expression of PRB were insignificant in the multivariate analysis (Table 5).

## Discussion

Using IHC techniques, we investigated the phosphorylation of ER-α Ser118 and ER-α Ser167, and expression of ER-α, ER-β1, ER-βcx/β2, PR, PRA, and PRB, in primary breast tumor specimens from 75 patients with metastatic breast cancer who, on relapse, received endocrine therapy as first-line treatment. Our results indicate that patients with primary breast tumors with high phosphorylation of ER-α Ser167, or high expression of ER-α, PR, PRA, or PRB, significantly respond to endocrine therapy and had a better survival after relapse.

ER-α is phosphorylated on multiple amino acid residues [3]. In general, phosphorylation of serine residues in the AF1 domain of ER-α appears to influence the recruitment of coactivators, resulting in enhanced ER-mediated transcription. In this study, we measured the phosphorylation of ER-α Ser118 and Ser167 as well as the expression of ER-α in breast cancer by IHC using site-specific anti-ER-α-phosphoserine antibodies. Our results showed that phosphorylation of ER-α Ser118, but not of ER-α Ser167, was significantly correlated with expression levels of HER2. It has been reported that ER-α was significantly phosphorylated on Ser118 in response to either estradiol binding or the activation of the mitogen-activated protein kinase (MAPK) pathway, while Ser167 is phosphorylated by AKT, p90 ribosomal S6 kinase (RSK), and casein kinase II as well as MAPK [5,7,9,24]. Murphy and colleagues recently reported that in 45 human breast tumor biopsies phosphorylation of ER-α Ser118 correlated with active MAPK [25]. Because MAPK is located downstream of HER2, it is possible that phosphorylation of ER-α Ser118 is in part caused by HER2-MAPK signaling in breast cancer. On the other hand, phosphorylation of ER-α Ser167 seems to be led by different mechanisms.

Our results also showed that while phosphorylation of both ER-α Ser118 and Ser167 was strongly and positively correlated with expression of ER-β 1 and ER-βcx/β2, there was no observed association between expression of ER-α and ER-β proteins. Both antibodies for ER-β1 and ER-βcx/β2, generated in this study, were specific against their respective C-terminal amino acid residues, and positive nuclear staining was observed in normal breast epithelial cells, noninvasive ductal carcinoma, and invasive carcinoma. Saunders and colleagues also found that there was no quantitative relation between IHC scores for ER-α and ER-β [26]. However, using IHC, it was reported that ER-β expression was positively correlated with ER-α and PR [27]. Specific detection of ER-β1 from other isoforms also indicated a positive correlation between expression of ER-β1 and ER-α [28]. However, no studies have been reported concerning the relation between phosphorylation of ER-α and expression of ER-β proteins.

In our analysis, ER-α expression was positively correlated with PRA but not with PRB. In addition, phosphorylation of both ER-α Ser118 and Ser167 was strongly and positively associated with expression of PRA but not with PRB. This suggests that PRA is preferentially induced following the transcription of ER-α after the phosphorylation of Ser118 and/or Ser167. Two previous studies have reported investigations into the expression of PRA and PRB in breast cancer. The first, an analysis of 202 PR-positive breast cancers by immunoblotting, showed that while there was no significant difference in levels of PRA and PRB in most of the PR-positive tumors, nevertheless expression levels of PRA were higher in 59% of tumors and at least four times as high in 25% [29]. In the second study, of 32 PR-positive breast cancers, it was reported that excess PRB correlated with the absence of HER2, thereby indicating a good prognosis, whereas excess PRA correlated with a poorly differentiated phenotype and higher tumor grade [30]. The normally equal expression of PRA and PRB is disrupted early in carcinogenesis. PRA is usually the predominant isoform in tumors of the breast, and it appears, therefore, that disrupted progesterone signaling may play a role in the development or progression of these cancers [14,29,31].

The most important results to come out of this study concern the correlation between clinical response and the phosphorylation and expression of the receptors. We identified that patients with primary breast tumors with high phosphorylation of ER-α Ser167, or high expression of PRA or PRB, significantly responded to endocrine therapy, whereas phosphorylation of ER-α Ser118 and expression of ER-β1 and ER-βcx/β2 did not influence response. Phosphorylation of both ER-α Ser118 and ER-α Ser167 occurs in response to either estradiol binding or activation of growth factor signaling pathways. It is well established that peptide growth factor signaling pathways can activate ER-α, in the absence of its ligand, through phosphorylation of ER-α by MAPK [8,32]. In addition, the induction of ER-α by MAPK also enhances ER signaling and promotes tumor growth in the presence of estradiol, and such tumors have been shown to be responsive still to antiestrogen therapy [33]. Furthermore, Clark and colleagues reported that, independently of MAPK, p90 ribosomal S6 kinase 2 (Rsk2) specifically activates ER-α by phosphorylation of Ser167 and by docking to the hormone-binding domain of ER-α, and that the antiestrogen 4-hydroxytamoxifen blocks Rsk2-mediated activation of ER-α [7]. Since our results showed that phosphorylation of ER-α Ser167, but not ER-α Ser118, was predictive of response to endocrine therapy, they suggest that, in breast cancer, phosphorylation of ER-α Ser118 occurs frequently without estradiol, whereas phosphorylation of ER-α Ser167 may occur frequently in response to estradiol binding.

It has been reported that HER2-induced MAPK and ER-α activation leads to tamoxifen resistance [34]. Data from these clinical trials demonstrated that the antiproliferative response to endocrine therapy was impeded in ER-α-positive/HER2-positive primary breast cancers [35]. In contrast, a Southwest Oncology Group study reported that overexpression of HER2 was not associated with tamoxifen unresponsiveness or a more aggressive phenotype of ER-α-positive metastatic breast cancer [36]. In our analysis, HER2-positive tumors showed high phosphorylation levels of ER-α Ser118 and were resistant to endocrine therapy.

Finally, our results showed that expression of ER-β1 and ER-βcx/β2 does correlate with response to endocrine therapy. No significant differences in the expression of ER-β1, ER-β2, and ER-β5 mRNAs between tamoxifen-sensitive and -resistant groups, has been reported [37]. Taken together, these data suggest that the expression of ER-β proteins is not predictive of response to endocrine therapy in breast cancer. However, a significant correlation between a PR-negative phenotype and the presence of ER-βcx/β2 in ER-α-rich tumor foci and expression of ER-βcx/β2 with low PR expression has been shown to correlate with a poor response to tamoxifen [38].

In our analysis, the expression of PRA and PRB as well as PR was strongly predictive of response to endocrine therapy. In contrast, it has been reported, in a study of T47D human breast tumor xenografts, that tamoxifen treatment preferentially inhibited the growth of PRA tumors, whereas PRB tumors were unaffected [39]. Another study reported that, although estrogen induced PR expression in all breast cancer cell lines studied, the expression ratio of PRA/PRB induced by estrogen was dependent on the cell line, and that these results suggested that the PRA and PRB promoters were differentially regulated by estrogen in different breast cancer cells [40]. Further studies are obviously needed to resolve these apparent discrepancies and in order to identify the functional importance of altered PR isoform expression and how this might affect the response of breast tumors to endocrine therapy.

## Conclusion

The present study has shown for the first time that patients with primary breast tumors with either high phosphorylation of ER-α Ser167 or high expression of PRA or PRB respond significantly to endocrine therapy and have a better survival after relapse. Our data suggest that phosphorylation of ER-α Ser167 is helpful in selecting patients who may benefit from endocrine therapy and is a prognostic marker in metastatic breast cancer.

## Abbreviations

AF = action function; DMEM = Dulbecco's modified essential medium; E_2 _= 17β-estradiol; EGF = epidermal growth factor; ER = estrogen receptor; IHC = immunohistochemistry/immunohistochemical; MAPK = mitogen-activated protein kinase; PR = progesterone receptor.

## Competing interests

The authors declare that they have no competing interests.

## Authors' contributions

HY conceived of the study and participated in its design, coordination, and manuscript writing. MN carried out immunostaining experiments. SK, YF, and HI participated in its design and coordination and helped to draft the manuscript. YA, HS, MH, and KM provided tissue samples. ZZ assessed the immunostaining. All authors read and approved the final manuscript.

## Figures and Tables

**Table 1:** Clinicopathological characteristics of patients with metastatic breast cancer, their primary tumors, and treatment

Characteristic	Number of patients
Total number of patients	75
Age at diagnosis (years)	
≤50	35
>50	40
Range	29 to 77
Tumor size (cm)	
<2.0	20
≥2.0	55
Number of positive lymph nodes	
0	21
1–3	21
>3	33
Histological grade	
1	12
2	43
3	20
HER2	
Negative	63
Positive	12
Adjuvant therapy	
None	5
Endocrine therapy	32
Chemotherapy	2
Combined	36
Disease-free interval (months)	
Mean ± standard deviation	39.9 ± 26.4
Median	38
Range	1 to 123
First-line endocrine therapy	
Tamoxifen	56
Aromatase inhibitors	11
LH-RH agonist	3
LH-RH agonist + tamoxifen	4
Fulvestrant	1

LH-RH agonist, luteinizing hormone–releasing hormone agonist.

**Table 2:** Correlations between immunohistochemistry scores for expression and phosphorylation of hormone receptors in primary breast tumors

Receptor	ER-α (1D5)	ER-α Ser118	ER-α Ser167	ER-β1	ER-βcx/β2	PR (636)	PRA
ER-α Ser118	+0.224^a^						
	0.055^b^						
ER-α Ser167	+0.140	+0.556					
	0.23	<0.0001*					
ER-β1	+0.039	+0.589	+0.417				
	0.74	<0.0001*	0.0003*				
ER-βcx/β2	+0.009	+0.640	+0.518	+0.693			
	0.94	<0.0001*	<0.0001*	<0.0001*			
PR (636)	+0.446	+0.032	+0.011	+0.004	-0.015		
	0.0001*	0.78	0.92	0.97	0.90		
PRA	+0.256	+0.463	+0.394	+0.203	+0.378	+0.500	
	0.028*	<0.0001*	0.0007*	0.081	0.0011*	<0.0001*	
PRB	+0.187	+0.211	+0.149	+0.206	+0.325	+0.553	+0.526
	0.11	0.070	0.20	0.077	0.0052*	<0.0001*	<0.0001*

^a^Spearman correlation coefficient; ^b^*P*, Spearman rank correlation test. * *P *<0.05.

ER, estrogen receptor; PR(A,B), progresterone receptor (A,B).

**Table 3:** Correlation between immunohistochemistry scores for hormone receptors and response to endocrine therapy in breast cancer

Receptor	Responders (*n *= 35)^a^	Nonresponders (*n *= 40)^a^	*P*^b^	*P*^c^
ER-α (1D5)	5.8 ± 2.3 (7; 0–8)	4.1 ± 2.9 (5; 0–8)	0.0045*	0.0046*
ER-α Ser118	4.3 ± 2.6 (5; 0–8)	4.2 ± 2.4 (4; 0–8)	0.90	0.96
ER-α Ser167	2.5 ± 2.0 (2; 0–6)	1.6 ± 1.7 (2; 0–5)	0.033*	0.025*
ER-β1	4.2 ± 2.2 (4; 0–8)	4.5 ± 2.3 (5; 0–8)	0.43	0.57
ER-βcx/β2	3.1 ± 2.4 (3; 0–8)	3.0 ± 2.5 (2; 0–8)	0.72	0.80
PR (636)	5.5 ± 2.5 (6; 0–8)	3.6 ± 2.7 (4; 0–8)	0.0008*	0.0014*
PRA	4.6 ± 2.0 (5; 0–8)	2.4 ± 2.5 (2; 0–8)	0.0001*	<0.0001*
PRB	4.0 ± 2.1 (4; 0–8)	2.7 ± 2.4 (3; 0–8)	0.013*	0.015*

^a^Values are means ± standard deviations (medians; ranges). ^b^Mann–Whitney *U *test. ^c^Unpaired *t*-test. *P <0.05. ER, estrogen receptor; PR(A,B), progresterone receptor (A,B).

**Table 4:** Correlation between immunohistochemistry scores for hormone receptors and disease-free interval

Receptor	Spearman correlation coefficient	Spearman rank correlation test (*P*)
ER-α (1D5)	+0.246	0.035*
ER-α Ser118	+0.083	0.47
ER-α Ser167	+0.310	0.0076*
ER-β1	+0.039	0.74
ER-βcx/β2	+0.101	0.39
PR (636)	+0.276	0.018*
PRA	+0.319	0.0061*
PRB	+0.263	0.023*

*P <0.05. ER, estrogen receptor; PR, progresterone receptor.

**Table 5:** Comparison of immunohistochemistry scores for hormone receptors in primary and secondary tumors

Receptor	Primary tumors^a^	Secondary tumors^a^	*P*
ER-α (1D5)	4.9 ± 2.7	3.1 ± 3.2	0.049*
ER-α Ser118	4.9 ± 2.8	7.3 ± 1.6	0.0098*
ER-α Ser167	3.7 ± 1.6	5.5 ± 2.2	0.11
ER-β1	5.5 ± 2.2	7.0 ± 2.0	0.041*
ER-βcx/β2	4.1 ± 2.6	6.1 ± 2.6	0.049*
PR (636)	4.1 ± 3.2	2.9 ± 3.5	0.057
PRA	3.5 ± 2.6	6.1 ± 2.6	0.018*
PRB	3.7 ± 2.4	5.7 ± 2.6	0.027*

^a^Mean ± SD. *n *= 10. *P <0.05. ER, estrogen receptor; PR(A,B), progresterone receptor (A,B).

**Table 6:** Univariate and multivariate analysis of factors predicting postrelapse survival

	*Univariate*			*Multivariate*		
	
Factor	*P*	RR	95% CI	*P*	RR	95% CI
Age	0.67	0.882	0.494–1.576			
Tumor size	0.21	1.483	0.797–2.759			
Lymph node status	0.40	0.746	0.376–1.479			
Histological grade	0.20	0.542	0.213–1.382			
HER2	0.0033*	0.340	0.166–0.699			
ER-α (1D5)	0.0009*	2.924	1.550–5.515	0.0076*	2.465	1.271–4.781
ER-α Ser118	0.36	1.308	0.732–2.340			
ER-α Ser167	0.035*	1.890	1.045–3.420	0.19	1.536	0.841–2.900
ER-β1	0.37	1.355	0.697–2.635			
ER-βcx/β2	0.10	1.627	0.905–2.926			
PR (636)	0.0012*	2.796	1.498–5.220			
PRA	0.03*	2.182	1.202–3.960			
PRB	0.013*	2.157	1.177–3.951	0.24	1.499	0.763–2.945

CI, confidence interval; ER, estrogen receptor; PR, progesterone receptor; RR, relative risk. *P < 0.05.
